# Biofilm-forming strains of *P. aeruginosa* and *S. aureus* isolated from cystic fibrosis patients differently affect inflammatory phenotype of macrophages

**DOI:** 10.1007/s00011-023-01743-x

**Published:** 2023-05-31

**Authors:** Marta Ciszek-Lenda, Grzegorz Majka, Maciej Suski, Maria Walczewska, Sabina Górska, Edyta Golińska, Angelika Fedor, Andrzej Gamian, Rafał Olszanecki, Magdalena Strus, Janusz Marcinkiewicz

**Affiliations:** 1 Department of Immunology, Jagiellonian University Medical College, Faculty of Medicine, Czysta 18, 31-121 Krakow, Poland; 2 Department of Pharmacology, Jagiellonian University Medical College, Faculty of Medicine, Grzegorzecka 16, 31-53 Krakow, Poland; 3 Department of Microbiology, Laboratory of Microbiome Immunobiology, Hirszfeld Institute of Immunology and Experimental Therapy, Polish Academy of Sciences, Weigla 12, 53-114 Wroclaw, Poland; 4 Department of Microbiology, Jagiellonian University Medical College, Faculty of Medicine, Czysta 18, 31-121 Krakow, Poland; 5 Department of Immunology of Infectious Diseases, Laboratory of Medical Microbiology, Hirszfeld Institute of Immunology and Experimental Therapy, Polish Academy of Sciences, Weigla 12, 53-114 Wroclaw, Poland

**Keywords:** Cystic fibrosis, Inflammation, *P. aeruginosa*, *S. aureus*, M1 macrophages

## Abstract

**Objective:**

Lung cystic fibrosis (CF) is characterized by chronic infections and hyperinflammatory response of neutrophils and macrophages. *P. aeruginosa* (PA) and *S. aureus* (MSSA, MRSA) are major pathogens of advanced CF. The main goal of this study was to compare the inflammatory phenotype of murine C57BL/6 macrophages exposed to PA57 with that exposed to MSSA60, both strains isolated from the same patient with severe CF. In the present study, we used C57BL/6 mice sensitive to lung infection with *P. aeruginosa.*

**Methods:**

We measured the release of cytokines and the expression of phenotypic markers of murine neutrophils and macrophages exposed to bacterial cells and biofilm components (i.e., EPS) of the selected bacteria. In addition, a quantitative proteomic approach was used for the characterization of proteome-wide changes in macrophages.

**Results:**

Neutrophils stimulated with PA57 and MSSA60 strains produced hyperinflammatory pattern of cytokines. The pro-inflammatory impact of PA57 was significantly higher than that of MSSA60 (IL-6/IL-10 ratio: PA57 = 9.3 vs. MSSA60 = 1.7). Macrophages produced significantly lower amount of cytokines, but showed classical pattern of M1 markers (iNOS-High; arginase-1 and mannose receptor MRC1-Low). Importantly, as evidenced by proteomic analysis, PA57 and PA57-EPS were stronger inducers of M1 macrophage polarization than the MSSA60 counterparts.

**Conclusions:**

Our study demonstrated that strong biofilm *P. aeruginosa* strains, CF isolates, are dominant inducers of M1 macrophages, termed biofilm-associated macrophages (BAMs). We suggest that repolarization of detrimental BAMs might be a new therapeutic strategy to ameliorate the airway damage in CF.

**Supplementary Information:**

The online version contains supplementary material available at 10.1007/s00011-023-01743-x.

## Introduction

Cystic fibrosis (CF) is an autosomal recessive disorder caused by mutations in the gene encoding cystic fibrosis transmembrane conductance regulator (CFTR) responsible for the production of highly viscoelastic mucus and defective mucociliary clearance which facilitates bacterial trapping and formation of bacterial biofilms. Moreover, dysfunction of CFTR induces damage to all tissues lined with epithelial cells and mucus, including lungs. This leads to chronic inflammation generated by a failure to clear microorganisms and development of pro-inflammatory local microenvironment [1, 2]. Early in life, cystic fibrosis patients are infected with various pathogens, originally with *Staphylococcus aureus* (SA) strains, followed by *Pseudomonas aeruginosa* (PA) mucoid, biofilm-forming strains [3]. Moreover, during chronic and recurrent lung infections of CF, evolutionary diversification of PA has been observed which has been associated with PA strains’ phenotypic adaptation (e.g., auxotrophy, loss of virulence factors, and antibiotic resistance) [4].

On the other hand, according to our knowledge, the capacity of these bacteria and biofilm components to stimulate inflammatory cells has not been fully elucidated. It seems to be of great importance as chronic biofilm-associated infections of CF airways are accompanied by a massive infiltration of neutrophils, the major source of inflammatory mediators [5]. Both partners of the inflammatory response, pathogenic bacteria as inducers, and neutrophils as responders, contribute to the pathogenesis of lung CF. Namely, activated neutrophils fail to effectively clear the pathogens hidden in a biofilm. Instead, their activation leads to the accumulation of neutrophil elastase and reactive oxygens species (ROS) that degrade the airway matrix structural proteins and induce oxidative stress [6]. Finally, all these changes exacerbate and maintain chronic inflammation, reduce bacterial clearance, and destroy lung tissue integrity [7]. Recently, we have shown that neutrophils, termed as biofilm-associated neutrophils (BANs), exposed in vitro to the components of *P. aeruginosa* biofilm, produce a hyperinflammatory set of neutrophil-derived mediators [8].

Importantly, in contrast to the widely documented detrimental role of neutrophils in the pathogenesis of chronic CF, the role of macrophages is less ascertained [9]. Much as the impaired phagocytic and enhanced inflammatory properties of CF macrophages have been reported, the impact of major CF pathogens, *P. aeruginosa* and *S. aureus,* on these activities has not been determined [10]. Therefore, to clarify this problem, the following studies were performed:

1. Comparison of the inflammatory properties of neutrophils and macrophages stimulated by PA with that of MSSA/MRSA strains. Moreover, the impact of the PA strain isolated from the patient with severe CF was compared with that of the PA strain originating from the mild CF patient. All the tested bacteria were selected from biofilm-forming bacterial strains isolated from the sputum of CF patients with recurrent airways infections [11]. The origin of the bacteria is the advantage of this study, as *P. aeruginosa* strains isolated from patients with advanced CF are phenotypically different from those isolated at the early stages of the disease [12]. As target cells, we used peritoneal exudate cells derived from C57BL/6 mice sensitive to *P. aeruginosa* pulmonary infections [13, 14].
2. Verification of our hypothesis suggesting that bacteria that create the biofilm environment, such as those that exist in the CF airway, are responsible for the hyperinflammatory response of macrophages (M1-like) termed herein as cystic fibrosis-biofilm-associated macrophages (CF-BAM).

## Materials and methods

### Bacteria

All experiments in this study were performed using two *P. aeruginosa* strains coded as PA57 and PA43 and two *S. aureus* strains (MSSA60, MRSA75). Bacteria were isolated from the sputum of patients during an exacerbation of the advanced stage of CF. Criteria for selecting the strains were strong or weak ability to produce biofilm in comparison to other strains of the same species. As for the PA57 strain, it was isolated from a patient with a severe form of the disease (FEV1 = 13%) [11], had a strong biofilm production capacity, and in addition a very large amount of mucus observed on the agar around the colony. PA43 was originated from a patient with mild disease, produced a weak biofilm, and had no mucus around the colony observed on the agar plates. Out of *S. aureus* strains, we chose MSSA60 having strong biofilm production capacity, which was isolated from a patient with a severe form of the disease (the same from which PA57 was isolated), and MRSA75 originated from a patient with mild disease, with relatively weak ability to produce biofilm. Isolated bacteria were cultured in tryptic soy broth (TSB, Oxoid/Thermo Fisher Scientific, Fremont, CA, USA) for 72 h at 37 °C under aerobic conditions. After cultivation, bacteria were centrifuged for 10 min at 500* g* and washed with 10 ml of phosphate-buffered saline (PBS, pH 7.4, Sigma-Aldrich, Steinheim, Germany), killed, and in such form used for in vitro tests with immune cells. In some cases, bacteria cultures were used for isolations of biofilm matrix components (see below).

### Killing *P. aeruginosa* and *S. aureus* bacterial cells

Bacteria pellets originating from 72 h cultures were treated thrice with high temperature (121 °C) at 0.3 bars in the ASVE-ELMI ESS-207 SMS steam sterilizer and in that form used to stimulate immune cells (see below). The follow-up bacteria culture was verified to be sterile.

### Isolation of biofilm matrix components

#### DNA extraction

The 72 h bacterial culture was centrifuged (8000 rpm, 10 °C, 15 min). The pellet was washed twice by PBS and then incubated in 0.25 mL of 10 mM Tris–HCl (pH 8) and 2.5 mg/mL of lysozyme at 37 °C for 2 h. Then, 0.5 mL of lysis buffer (50 mM Tris, 100 mM EDTA, 1% SDS, pH 8) and 1 mg/mL of proteinase-K were added and incubated at 50 °C for 2 h in a water bath. The digestion with proteinase-K was followed by the addition of 0.5 mL of phenol:chloroform (1:1). The samples were mixed gently for 3 min and centrifuged (14 000 rpm, 4 °C, 15 min). The upper layer was transferred to a fresh tube and extracted with chloroform:isoamyl alcohol (24:1) by centrifugation at (14 000 rpm, 4 °C, 15 min). This step was repeated. The supernatant was precipitated with double volume of ethanol and left till the precipitate was formed. DNA was collected by centrifuging at 14 000 rpm, 4 °C, for 15 min, and dried. The pellet was suspended in 10 mM Tris–HCl (pH 8) and 1 mM EDTA (pH 8) buffer and incubated at 45 °C in a water bath for 3 h. The quality of DNA was checked using DS-11 spectrophotometer (DeNovix, Wilmington, DE, USA).

#### Lipopolysaccharide (LPS) isolation

LPS was isolated using hot phenol/water method and purified as described by Westphal et al. [15]. The quantity of LPS was measured after the sample lyophilization.

#### Exopolysacchairde (EPS) isolation

EPS was isolated and purified as described by Górska et al. [16]. Briefly, polysaccharide was obtained by trichloroacetic acid extraction of bacterial mass, precipitated with ethanol, and purified by DNAse, RNAse, and protease. EPS was purified by ion-exchange chromatography. The fractions containing neutral EPS were pooled, desalted by dialysis against water at 4 °C for 24 h, and lyophilized. Total saccharide concentration was measured by phenol sulfuric acid method according to Dubois’s method [17].

#### Peptidoglycan (PG) isolation

The isolation protocol was conducted according to the modified method of Schaub and Dillard [18]. Briefly, the bacterial mass was suspended in 25 mM phosphate buffer (pH 6) and added drop by drop to a boiling 8% SDS. Then, the suspension was incubated at 100 °C (30 min), cooled, and ultracentrifuged (45 000 rpm; 15 °C; 30 min). The obtained pellet was extracted one more time, washed (4–6 times) with phosphate buffer, and freeze-dried. To obtain pure PG, the freeze-dried mass underwent digestion by DNase, RNase, and protease.

### Mice

Inbred C57BL/6 mice (8–12 weeks of age, 18–22 g) were maintained at the Animal Breeding Unit of the Department of Immunology of Jagiellonian University Medical College. All mice were held in standard caging conditions with water and standard diet ad libitum.

### Isolation of peritoneal exudate cells

Peritoneal mouse exudate cells were induced by an intraperitoneal injection of 1.5 mL of 3% thioglycollate (Sigma-Aldrich). After 18 h (neutrophils) or 96 h (macrophages), mice were euthanized by overdosing of isoflurane vapors (Abbott Laboratories, Chicago, IL, USA) and cervical dislocation was performed. Cells were then collected by washing out the peritoneal cavity with 5 mL of PBS (Lonza, Verviers, Belgium) containing 5 U heparin/mL (Polfa, Warsaw, Poland). Cells were centrifuged, and red blood cells were lysed. Osmolarity was restored by the addition of PBS. At least three mice were used as donors of peritoneal exudate cells for each experiment.

### Cell viability

Cell viability was monitored by means of LDH activity (lactate dehydrogenase) using LDH assay kit (Thermo Fisher Scientific, Rockford, IL, USA) according to manufacturer’s instruction. The viability of phagocytes was controlled in all experimental systems to avoid cytotoxic effect of the tested agents.

### Stimulation of neutrophils and macrophages with bacterial products

Neutrophils and macrophages were cultured in 24-well flat-bottom cell culture plates at 5 × 10^5^/well in IMDM medium (Lonza) supplemented with 5% fetal bovine serum (FBS; Lonza), 2 mM stable L-glutamine (Lonza), and 50 mg/mL gentamicin (KRKA, Warsaw Poland) at 37 °C in an atmosphere of 5% CO_2_. To determine the influence of selected bacteria and their components on innate immune cell activity, neutrophils and macrophages were stimulated with heat-killed whole bacterial cells (20:1 bacteria per cell), or with their antigens (EPS, LPS, DNA, and PG at selected concentrations, see Figs. 3 and 4 captions). As a reference stimulus, we used 0.1 µg/mL LPS from *Escherichia coli* strain 0111:B4 (LPS, Sigma-Aldrich). After 24 h of stimulation, culture supernatants were collected and frozen at − 80 °C until use. Cell lysates were used for western blot analysis.

### Cytokine measurement

Cytokine levels in cell culture supernatants were measured by sandwich ELISA. Microtiter plates (Costar EIA/RIA plates, Corning) were coated with a cytokine-specific antibody. The expression levels of IL-6, IL-10, and IL-12p40 were measured according to the manufacturer’s instructions (OptEIA Sets, BD Biosciences). TNF-α level was measured according to the manufacturer’s instructions (ELISA uncoated kits, Invitrogen, Waltham, MA, USA). In all cases, 10% FBS in PBS was used as a blocking solution. TMB substrate solution (Invitrogen) was used to develop a colorimetric reaction, which was stopped with 2 M sulfuric acid. Optical density was measured at 450 (570) nm using a microtiter plate reader (PowerWaveX, Bio-Tek Instruments, Winooski, VT, USA).

### Nitric oxide (NO) determination

NO levels in culture supernatants of macrophages were quantified by the accumulation of nitrite as a stable end product, according to a modified Griess method [19]. Cell culture supernatant (100 µL) was mixed with 14 mM 4,4′-diamino-diphenylsulfone (Dapsone, Sigma-Aldrich) in 2 M HCl (50 µL) and 0.1% *N*-1-naphthylenediamine dihydrochloride (50 µL) in deionized water. The absorbance of the tested culture supernatants at 550 nm was compared with a sodium nitrate standard (NaNO_2_) curve.

### Chemiluminescence/ROS production

The effect of killed bacteria (PA57, PA43 MSSA and MRSA) on the production of ROS by neutrophils was evaluated in vitro using luminol-dependent chemiluminescence. Chemiluminescence was counted at 37 °C in a temperature-stabilized luminometer (Luminoscan, Thermo Fisher Scientific). Briefly, 18 h peritoneal cells induced by thioglycolate (5 × 10^5^/well) were mixed with luminol (0.8 mg/mL) in 1:1 volume ratio (both Sigma-Aldrich) on a 96-well flat-bottom black plate and incubated at 37 °C for 30 min (Thermo Fisher Scientific). After incubation, the cells were quickly stimulated with bacteria in 1:20 ratio (neutrophil:bacteria) or yeast zymosan (200 µg/mL, Sigma-Aldrich) and photon emission was measured for 75 min with 3 min intervals. Results are expressed as relative light units (RLU) where photons were counted every 5 s.

### Western blot

The levels of iNOS and arginase-1 protein were determined using western blot technique. Macrophages were lysed in lysis buffer containing a mixture of protease inhibitors (PBS, Triton X-100, 10% SDS, Sigma-Aldrich). The total protein concentration in the lysates was determined using a bicinchoninic acid protein assay kit (Sigma-Aldrich). Samples containing equal amounts of protein (14 mg) were suspended in loading buffer in a 2:1 ratio and denatured for 4 min at 100 °C. Samples were applied to polyacrylamide gel with 10% SDS and separated electrophoretically in the Laemmli system using Mighty Small II apparatus (Amersham Biosciences, UK). Separated proteins were transferred to the nitrocellulose membrane (Bio-Rad, Hercules, CA USA) using Hoefer TE22 transfer equipment (Amersham Biosciences). After overnight incubation with protein blocking solution at 4 °C (3% skimmed milk), membranes were incubated for 2 h at RT with rabbit polyclonal anti-iNOS (Enzo Lifesciences, Farmingdale, NY, USA) or anti-arginase-1 antibodies (Invitrogen) and mouse monoclonal anti-β-actin antibodies (Sigma-Aldrich). Then, membranes were incubated for 2 h with secondary antibodies, anti-rabbit IgG (Sigma-Aldrich), and goat IgG conjugated with phosphatase alkaline (Sigma-Aldrich,) at RT. The bands were detected with alkaline phosphatase substrate BCIP/NBT (Sigma, St. Louis, MO, USA). Protein bands were scanned and analyzed using freeware Scion Image for Windows (Scion, Frederick, MD, USA). Results are presented as a ratio of optical density of protein to β-actin, which is constitutively expressed in cells.

### Proteomics

#### Sample preparation for LC–MS/MS analysis

Macrophages were lysed in 150 µL of lysis buffer (2% SDS, 50 mM DTT in 0.1 M Tris–HCl pH 7.6), vortexed, incubated in 95 °C for 5 min, and clarified by centrifugation at 14 000* g* for 30 min. Before protein digestion, the total protein concentration in collected lysates was determined by WF assay [20, 21]. Next, a volume containing 70 µg of total protein was transferred to Microcon-30 kDa centrifugal filter units (Merck, Darmstadt, Germany), denatured with 8 M urea in 0.1 M Tris–HCl, pH 8.5, and digested to peptides with a use of filter-aided sample preparation (FASP) protocol [20]. Briefly, proteins were alkylated with iodoacetamide and cleaved with LysC–trypsin mix (Thermo Scientific, Waltham, MA, USA) with the enzyme to protein ratio 1:50. Digestions were carried out overnight in 50 mM Tris–HCl, pH 8.5, at 37 °C. After digestion, the peptide yields were determined by WF assay and the aliquots containing equal amount of total peptides were desalted on 96-well MiniSpin C18 columns (Harvard Apparatus, Holliston, MA, USA). Samples were then concentrated to a volume of ~ 5 µL and stored at − 80 °C. For project-specific spectral libraries preparation, equal amount of peptides from 40 samples distributed across all experimental conditions were combined and subjected to the fractionation protocol. HpH fractionation on C18 Micro SpinColumns (Harvard Apparatus) was performed in 50 mM ammonium formate buffer (pH 10) with 13 consecutive injections of the eluent buffer, comprising 5, 10, 12.5, 15, 17.5, 20, 22.5, 25, 27.5, 30, 35, 50, and 80% acetonitrile in 50 mM ammonium formate buffer (pH 10), collected by centrifugation (300 × *g*, 2 min), and dried in a speedvac concentrator (Eppendorf, Hamburg, Germany). In this way, peptides were distributed across 13 HpH fractions and analyzed by LC–MS/MS in DDA acquisition mode for library generation. Prior to the analysis, all samples and library peptide fractions were solubilized in 0.1% formic acid in a concentration of 0.5 µg/µL and spiked with the iRT peptide mix (Biognosys, Schlieren, Switzerland) for normalization of the retention time.

#### Liquid chromatography–tandem mass spectrometry

Peptides (1 µg) were injected onto a nanoEase M/Z Peptide BEH C18 75 µm i.d. × 25 cm column (Waters, Milford, MA, USA) via a trap column nanoEase M/Z Symmetry C18 180 µm i.d. × 2 cm column (Waters). For library generation, each peptide fraction was separated using a 98 min 1% to 40% B phase linear gradient (A phase—0.1% FA; B phase—80% ACN and 0.1% FA) operating at a flow rate of 300 nL/min on an UltiMate 3000 HPLC system (Thermo Scientific) and applied to a TripleTOF 6600 + (Sciex, Framingham, MA, USA) mass spectrometer. The main working nano-electrospray ion source (Optiflow, Sciex, Framingham, MA, USA) parameters were as follows: ion spray voltage 3.2 kV, interface heater temperature (IHT) 200 °C, ion source gas 1 (GS1) 10, and curtain gas (CUR) 25. For DDA acquisition, spectra were collected in full scan mode (350–1400 Da), followed by 100 CID MS/MS scans of the 100 most intense precursor ions from the preceding survey full scan exceeding 100 cps intensity under dynamic exclusion criteria. Samples analyzed in SWATH acquisition mode were separated using a 63 min 1–40% B phase linear gradient at a flow rate of 300 nL/min. For SWATH acquisition, spectra were collected in full scan mode (400–1250 Da), followed by 100 SWATH MS/MS scans using a variable precursor isolation window approach, with m/z windows ranging from 6 to 90 Da.

#### Mass spectrometric raw data analysis, spectral library generation, and SWATH quantitation

DDA data were searched against the murine UniProt database (release 2021_01_04, 17 056 entries) using the Pulsar search engine implemented in Spectronaut 16 software (Biognosys) [22] with default parameters (± 40 ppm mass tolerance on MS1 and MS2 level, mutated decoy generation method, trypsinP enzyme specificity). Deep learning-assisted iRT regression was set as iRT reference strategy for RT to iRT calibration with minimum R2 set to 0.8. Peptide, protein and PSM FDR were set to 1%. Library was generated using 3–6 fragment ions per precursor.

Project-specific library was then used to analyze the SWATH data in Spectronaut 16 (Biognosys). Data were filtered by 1% FDR on peptide and protein level, while quantitation and interference correction were done on the MS2 level. Protein grouping was performed based on the ID picker algorithm [23]. Protein quantities were calculated by averaging the respective peptide intensities, while the latter were obtained as mean precursor quantities. The protein coefficients of variation (CVs) were calculated based on the summed intensities of their respective peptides. Data were normalized by global regression strategy, while statistical testing for differential protein abundance was done using *t* tests with multiple testing correction after Storey [24]. Statistically significant differences (*q* value < 0.05) with quantitative cutoff for absolute 1.5-fold change were considered as differentially regulated. The LC–MS data, library and Spectronaut project have been deposited at the ProteomeXchange Consortium via the PRIDE partner repository [25] with the dataset identifier PXD036521.

### Statistical analysis

Statistical significance of differences between groups was analyzed using one-way ANOVA, followed, if significant, by a Dunnett’s test or Tukey’s for post hoc comparison. Results are expressed as mean ± SEM values. A *P* value < 0.05 was considered statistically significant. Analysis was performed using GraphPad Prism v. 5.01 (GraphPad Software, Inc. San Diego, CA, USA). The exact statistical analysis is named in the relevant figure’s caption.

## Results

### The effect of in vitro stimulation of neutrophils and macrophages with PA57, PA43, MSSA60, and MRSA75 bacteria on cytokine production

**Fig. 1:**
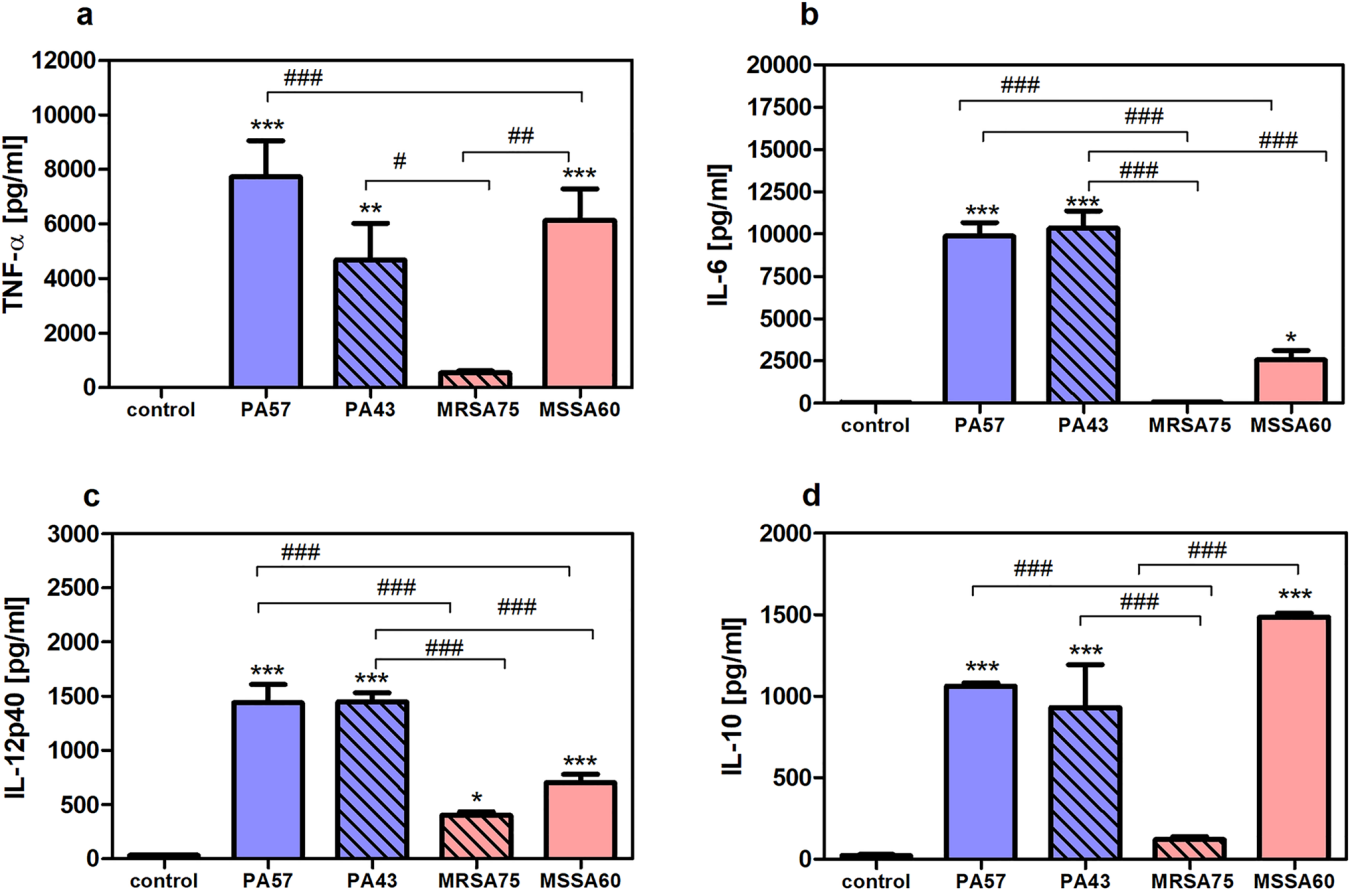
Secretory properties of neutrophils exposed to bacterial cells (PA57, PA43, MRSA75 and MSSA60). Levels of TNF-α (**a**), IL-6 (**b**), IL-12p40 (**c**), and IL-10 (**d**) were analyzed by ELISA of supernatants collected from 24 h cultures of neutrophils (5 × 10^5^/well). Data are mean ± SEM values of three independent experiments. Each group was run in triplicate. *p < 0.05, **p < 0.005, ***p < 0.001 vs. non-stimulated cells as negative control, one-way ANOVA and Dunnett's as post hoc comparison test. ^#^*p* < 0.05, ^##^*p* < 0.005, ^###^*p* < 0.001, one-way ANOVA and Tukey’s as post hoc comparison test

**Fig. 2:**
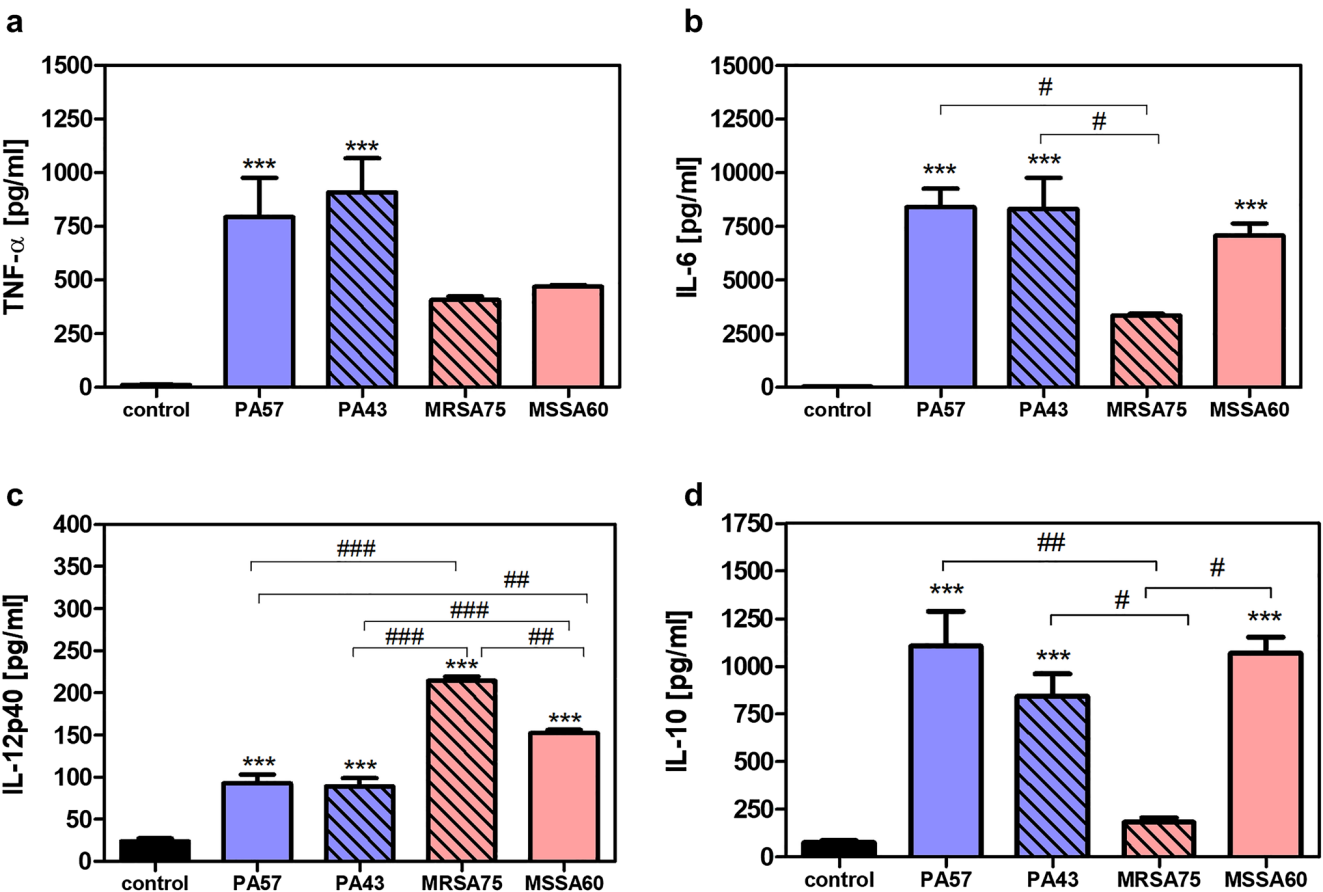
Secretory properties of macrophages exposed to bacteria cells (PA57, PA43, MRSA75 and MSSA60). Levels of TNF-α (**a**), IL-6 (**b**), IL-12p40 (**c**), and IL-10 (**d**) were analyzed by ELISA of the supernatants collected from 24 h cultures of macrophages. (5 × 10^5^/well). Data are mean ± SEM values of three independent experiments. Each group was run in triplicate. ***p < 0.001 vs. non-stimulated cells as negative control, one-way ANOVA and Dunnett's as post hoc comparison test. ^#^*p* < 0.05, ^##^*p* < 0.005, ^###^*p* < 0.001, one-way ANOVA and Tukey’s as post hoc comparison test

In this study, we examined the immunostimulatory potential of bacteria isolated from patients with CF. For this purpose, murine peritoneal neutrophils and macrophages were cultured with whole killed bacteria. Effective, non-cytotoxic, stimulatory amounts of bacteria (20:1) were chosen according to our previous work [8]. We observed that both pro-inflammatory (TNF-α, IL-6, IL-12p40) and anti-inflammatory cytokines (IL-10) were released from immune cells in response to dead bacteria (Figs. 1, 2). Furthermore, there were significant differences in the profile of released cytokines upon incubation with distinct bacterial strains. PA strains were much stronger inducers of cytokines secretion in comparison to SA strains. In addition, in most cases, the ratio of secreted IL-6 and IL-10 induced by PA strains was higher than by SA strains, which clearly indicates the pro-inflammatory pattern of cytokine release by cells stimulated with these bacteria (suppl. Table S1). Importantly, neutrophils secreted markedly higher amounts of pro-inflammatory cytokines than macrophages (e.g., TNF-α neutrophils/macrophages: PA57 ~ 7750/ ~ 790 pg/mL; MSSA60 ~ 6140/ ~ 470 pg/mL) (Figs. 1, 2).

### Stimulatory properties of biofilm components isolated from PA57, PA43, MSSA60, and MRSA75 bacteria cultures

**Fig. 3:**
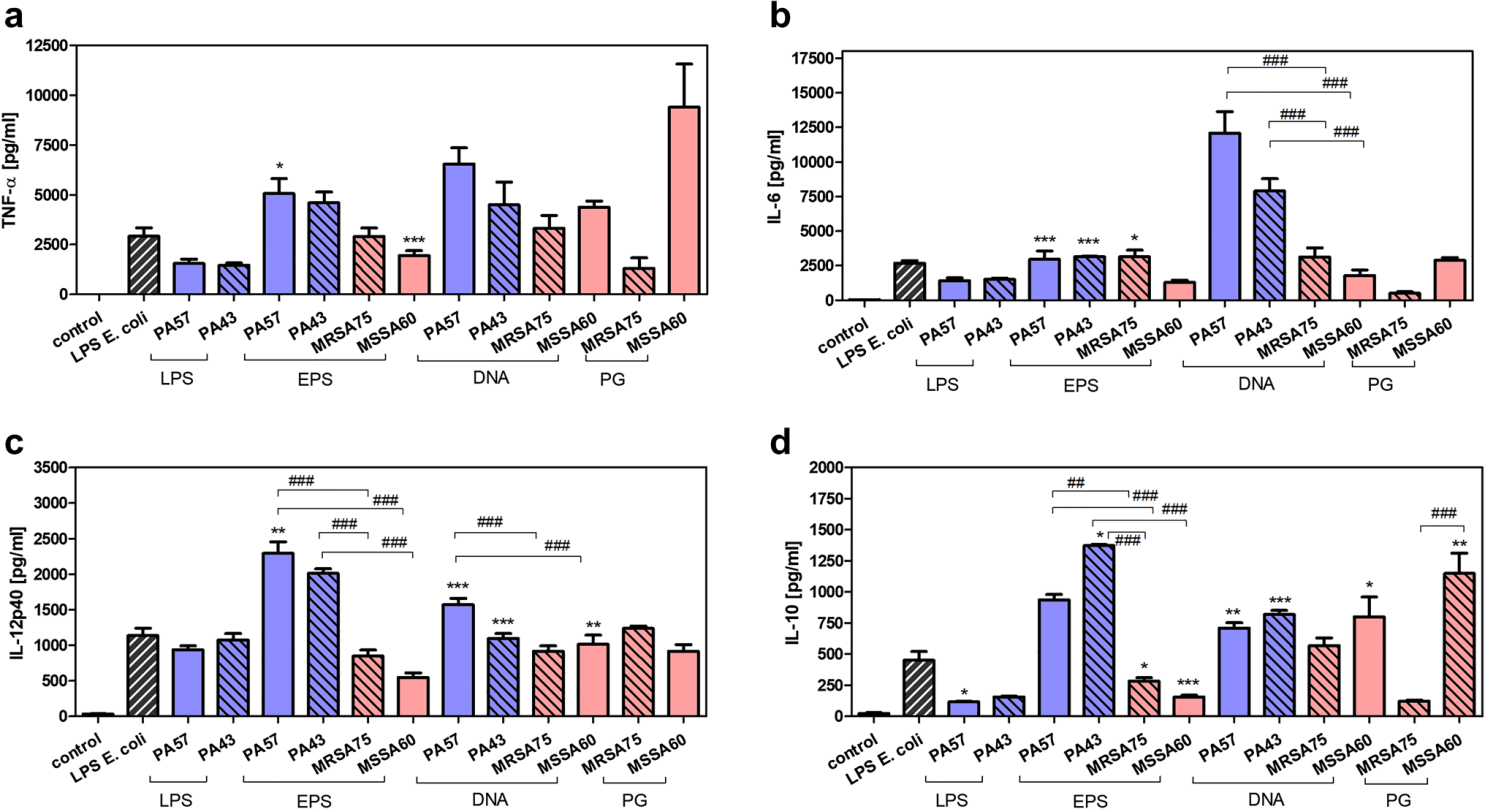
Cytokine production by neutrophils exposed to bacterial biofilm components (LPS 0.1, EPS 30, DNA 3, PG 10 µg/mL). The levels of TNF-α (**a**), IL-6 (**b**), IL-12p40 (**c**), and IL-10 (**d**) were analyzed by ELISA of supernatants collected from 24 h cultures of neutrophils (5 × 10^5^/well). Data are mean ± SEM values of three independent experiments. Each group was run in triplicate. *p < 0.05, **p < 0.005, ***p < 0.001 vs. LPS *E. coli* (0.1 µg/ml) as positive control, one-way ANOVA and Dunnett's as post hoc comparison test. ^##^*p* < 0.005, ^###^*p* < 0.001, one-way ANOVA and Tukey’s as post hoc comparison test

**Fig. 4:**
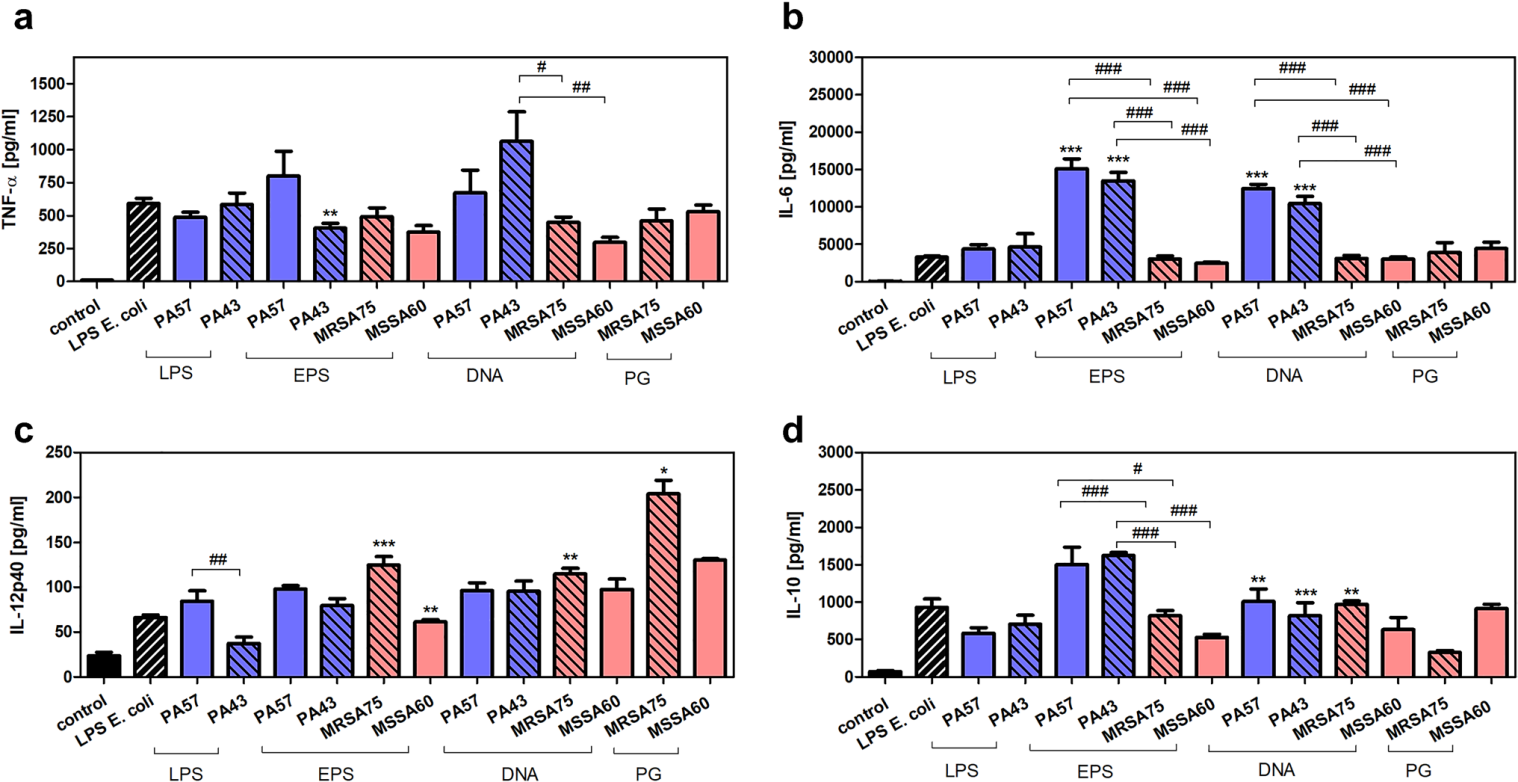
Cytokine production by macrophages exposed to bacterial biofilm components (LPS 0.1, EPS 30, DNA 3, PG 10—µg/mL). Levels of TNF-α (**a**), IL-6 (**b**), IL-12p40 (**c**), and IL-10 (**d**) were analyzed by ELISA of supernatants collected from 24 h cultures of macrophages (5 × 10^5^/well). Data are mean ± SEM values of three independent experiments. Each group was run in triplicate. *p < 0.05, **p < 0.005, ***p < 0.001 vs. LPS *E. coli* (0.1 µg/ml) as positive control, one-way ANOVA and Dunnett's as post hoc comparison test. ^#^*p* < 0.05, ^##^*p* < 0.005, ^###^*p* < 0.001, one-way ANOVA and Tukey’s as post hoc comparison test

Exopolysaccharide, DNA, and components of bacterial cell wall, such as LPS and PG, are the most abundant elements of biofilm matrix produced by the tested bacteria. The presence of biofilm, and hence of all these elements in CF, is significant. Assuming that in vivo cells infiltrating lungs have their first contact with biofilm, we decided to examine the effect of neutrophils’ and macrophages’ stimulation with this component on their cytokine secretion. Both the cells were incubated with the non-toxic, stimulatory concentration of all components which were chosen according to preliminary experiments measuring dose-dependent effects (data not shown). Their impact was compared with the effect of *E. coli* LPS, referential stimulator of inflammatory mediators. We observed that all tested agents markedly stimulated cytokine secretion from neutrophils and macrophages. However, the components isolated from MSSA60 and MRSA75 expressed lower stimulatory effect in comparison to PA57 and PA43 (Figs. 3, 4).

### The effect of stimulation of macrophages with PA57, PA43, MSSA60, and MRSA75 bacteria on iNOS and arginase-1 expression

**Fig. 5:**
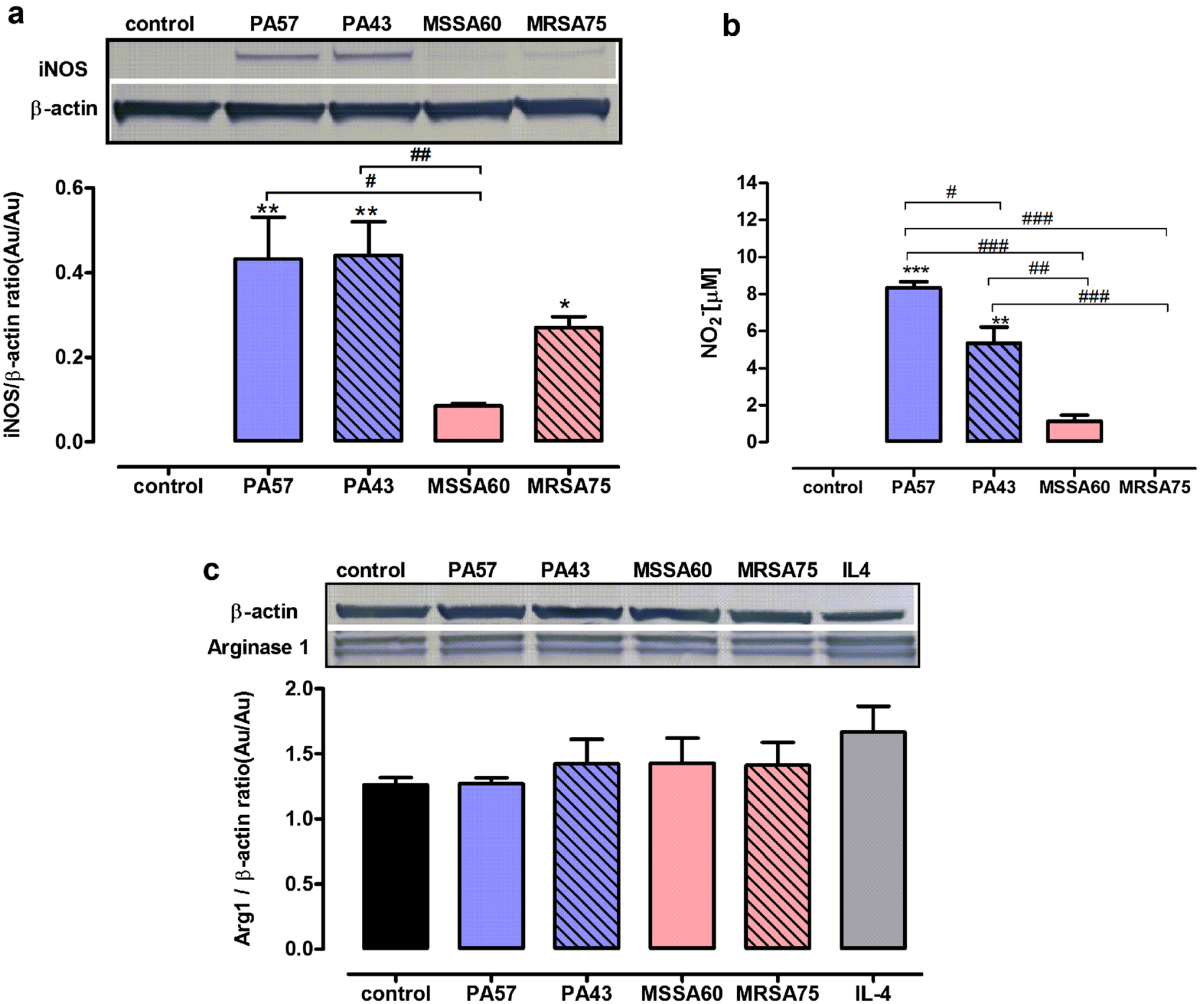
Expression of iNOS and arginase- proteins in macrophages exposed to bacteria cells (PA57, PA43, MSSA60, and MRSA75). Expression levels of arginase- (**a**) and iNOS (**b**) were measured by western blot analysis of cell lysates collected from 24 h cultures of macrophages (5 × 10^5^/well). Macrophages were incubated with tested bacteria at a ratio of 20:1. Representative western blot and densitometric analysis of bands from three experiments are shown. Data are normalized to constitutive expression levels of β-actin. *p < 0.05, **p < 0.005 vs. non-stimulated cells as negative control, one-way ANOVA, and Dunnett's as post hoc comparison test. #p < 0.05, ##p < 0.005, one-way ANOVA and Tukey’s as post hoc comparison test. In the same experiments, level of NO2 in supernatants were measured (**c**). Data are mean ± SEM values of three independent experiments. Each group was run in duplicate. **p < 0.005, ***p < 0.001 vs. non-stimulated cells as negative control, one-way ANOVA and Dunnett's as post hoc comparison test. ^#^*p* < 0.05, ^##^*p* < 0.005, ^###^*p* < 0.001, one-way ANOVA and Tukey’s as post hoc comparison test

We measured the relative expression of iNOS and arginase-1 which are canonic markers of M1 and M2 polarization, respectively. Both *P. aeruginosa* strains have markedly induced iNOS in the tested cells. On the contrary, MSSA60 failed to activate iNOS production, while MRSA75 incubation yielded intermediary levels of the enzyme (Fig. 5a). Expression of arginase-1 was not altered upon stimulation with the tested bacteria (Fig. 5c). Moreover, measurement of nitrites in the culture medium confirmed that production of NO by macrophages, which had been incubated with PA strains, was increased in comparison to both control cells and cells that were treated with killed SA (Fig. 5b). These results point to potential polarization of macrophages into the M1 phenotype after contact with antigens from CF-relevant pathogens.

### Impact of CF pathogens and their biofilm components on the macrophage proteome

**Fig. 6:**
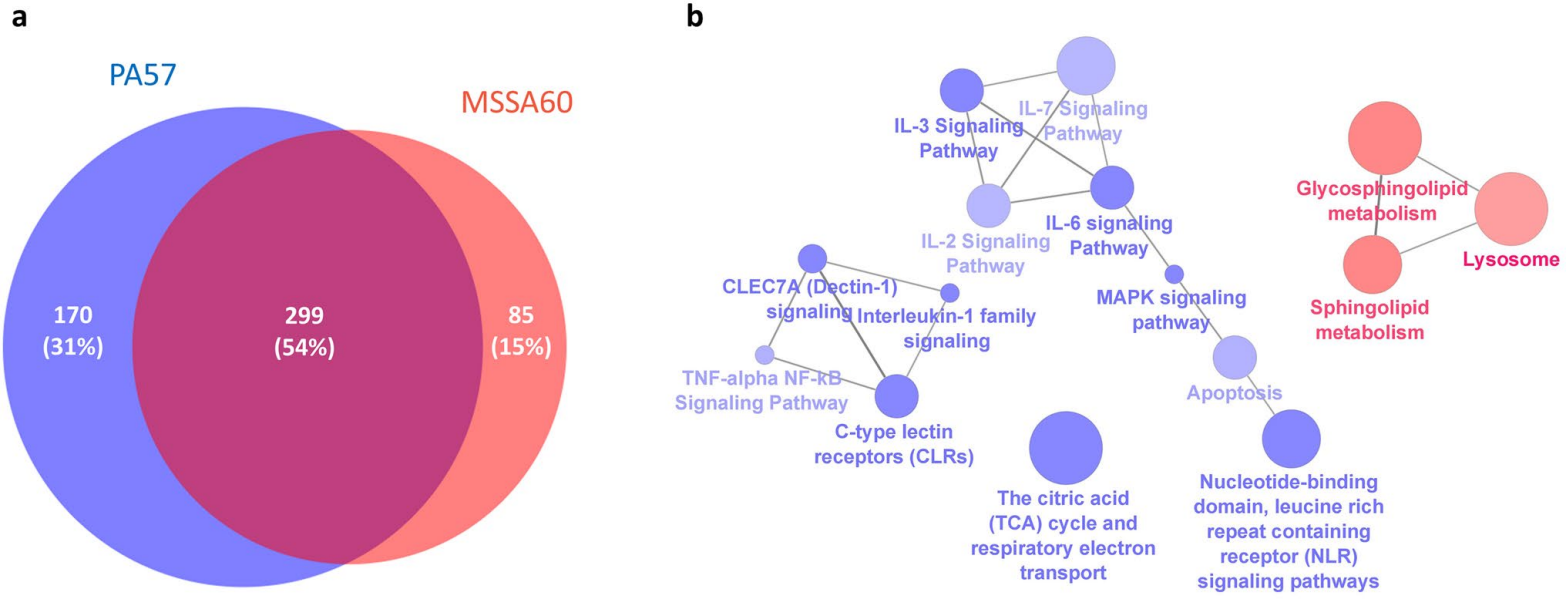
Proteome changes in PA57 and MSSA60-stimulated macrophages. The Venn diagram of the identified regulated proteins indicates that the changes elicited by the bacteria are qualitatively similar (**a**); however, quantitatively different and biased toward PA57 in terms of pro-inflammatory activation (**b**)

**Fig. 7:**
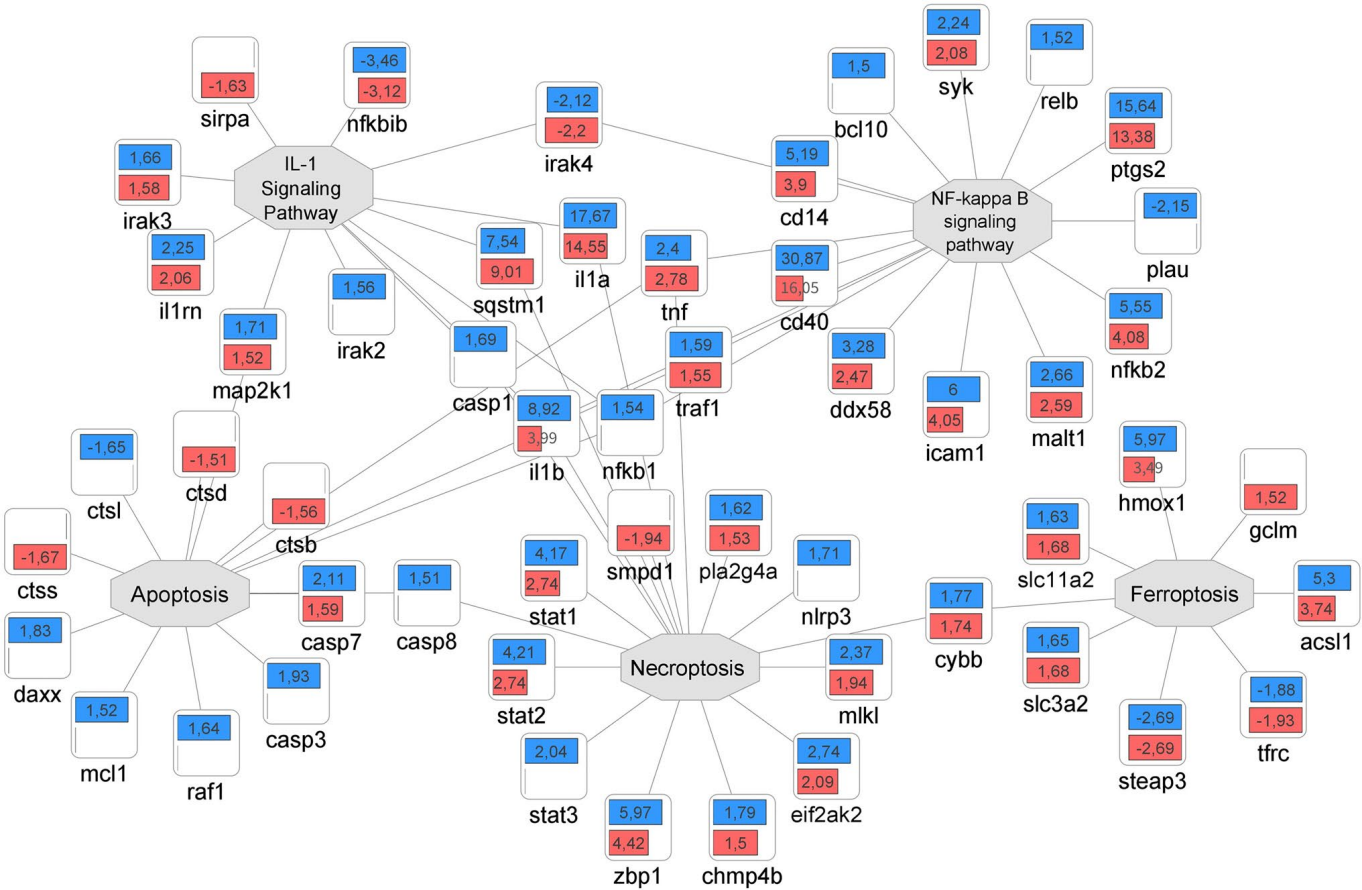
Quantitative comparison of the pro-inflammatory response elicited by PA57 and MSSA60 in macrophages. Fold change quantitative data indicate that PA57 (blue) induced more pronounced pro-inflammatory response as compared to MSSA60 (red), including several M1-like phenotype markers: NF-κB signaling, induction of cytokines and adhesion molecules, as well as intensification of apoptosis and necroptosis (color figure online)

**Fig. 8:**
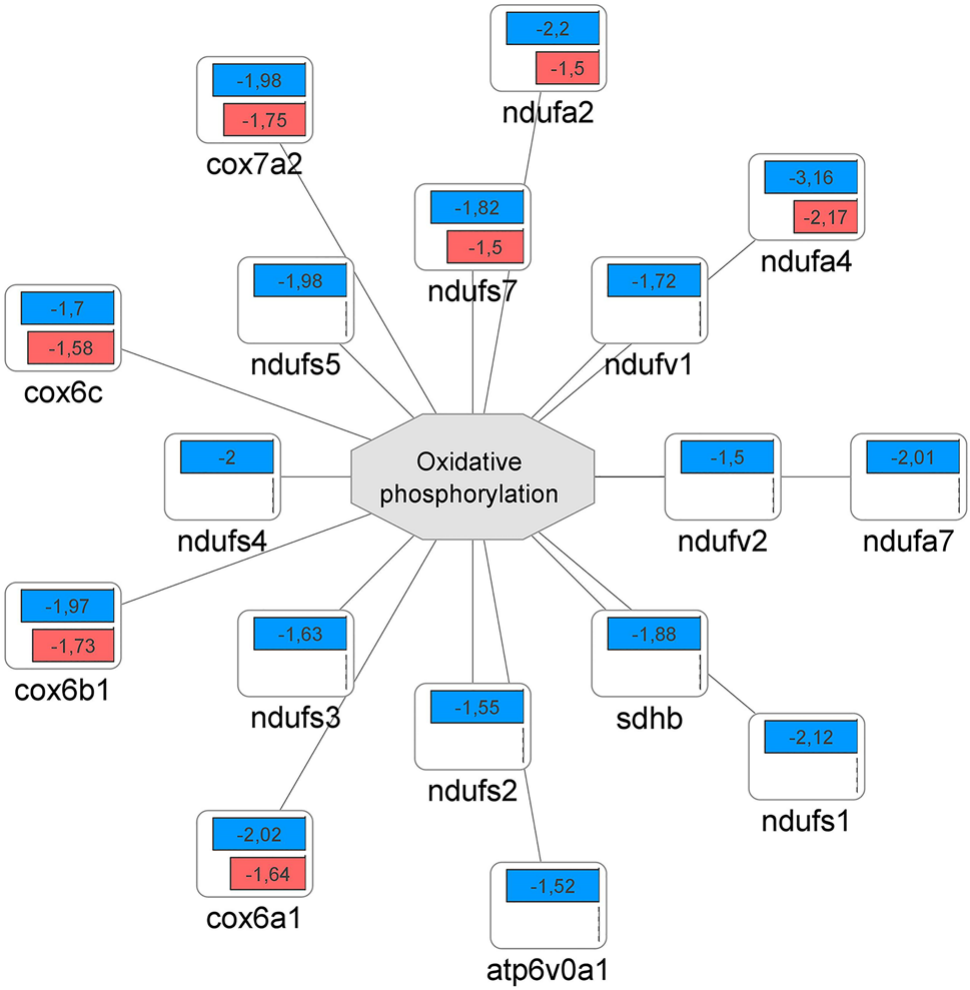
Mitochondrial oxidative phosphorylation repression by PA57 and MSSA60 bacteria in macrophages. Fold change quantitative data indicate, that PA57 (blue) repressed the abundance of oxidative phosphorylation-engaged proteins more significantly than MSSA60 (red) (color figure online)

**Fig. 9:**
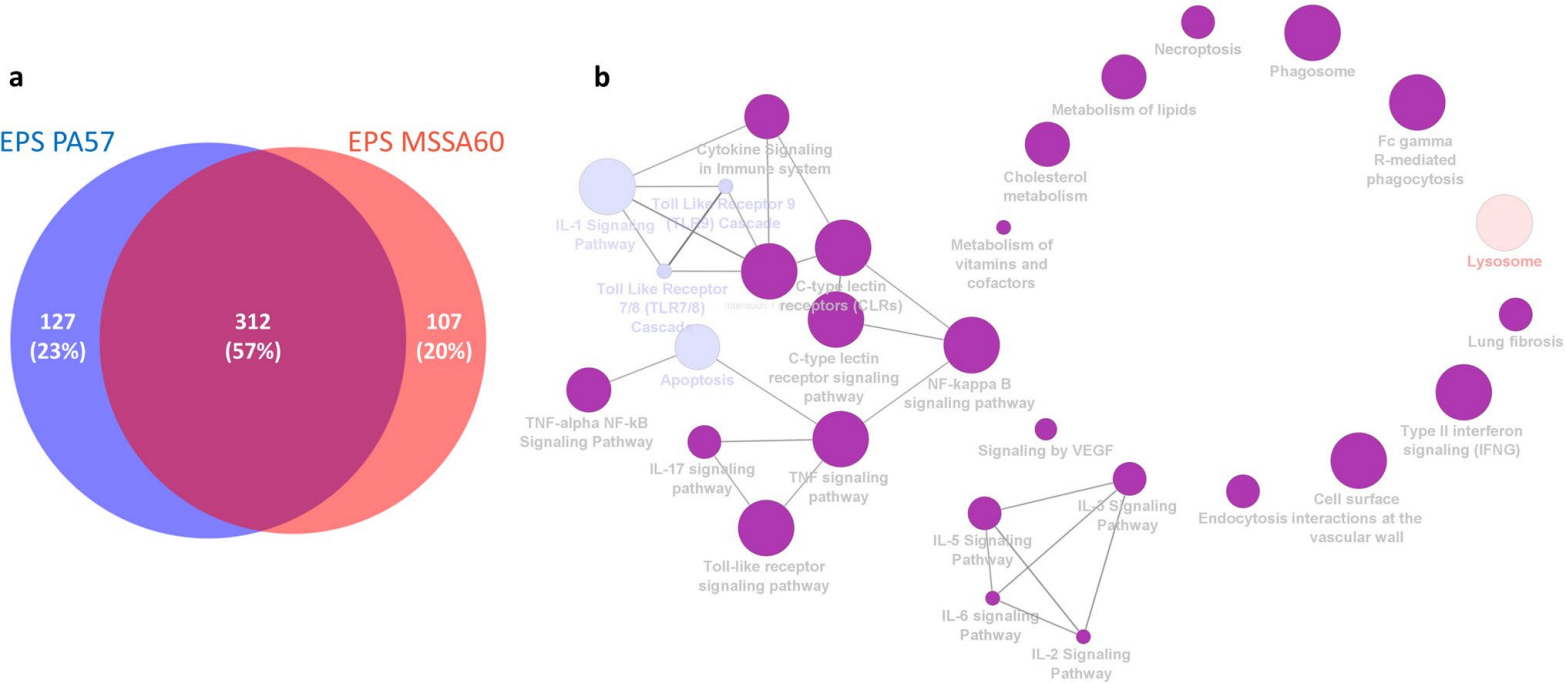
Proteome changes in exopolysaccharide-activated macrophages. The Venn diagram of the regulated proteins indicates that the changes elicited by bacterial exopolysaccharide extracted from PA57 and MSSA60 are qualitatively and quantitatively more similar than those originating from whole bacteria (**a**), however, biased toward PA57 in terms of pro-inflammatory activation (**b**)

DDA mass spectrometry measurements for the preparation of spectral libraries resulted in the identification of 64 191 proteotypic peptides, which translated into the number of 7 111 protein groups used as a SWATH search matrix with Spectronaut. Spectral library precursor recovery was 91.6%, while the completeness of the protein group data was 88.5%. The median CVs of the protein groups were calculated in the range of 15.6–21.0% for all experimental groups, which allowed for the estimation of a significant quantitative cutoff for an absolute 1.5-fold change (statistical power 99%). Quantitative comparisons with the control group revealed that 384, 469, 419, and 439 proteins were differentially regulated in the MSSA60 and PA57 groups, as well as those stimulated with EPS derived from MSSA60 and PA57, respectively (suppl. Tables S2–5). As expected, most of the identified proteins were functionally related to the initiation and propagation of the immune and inflammatory response with a numerous cytokine and chemokine signaling pathways regulated as compared to the control group (Figs. 6, 7, 8, 9 and Suppl. Fig. S1).

**Fig. 10:**
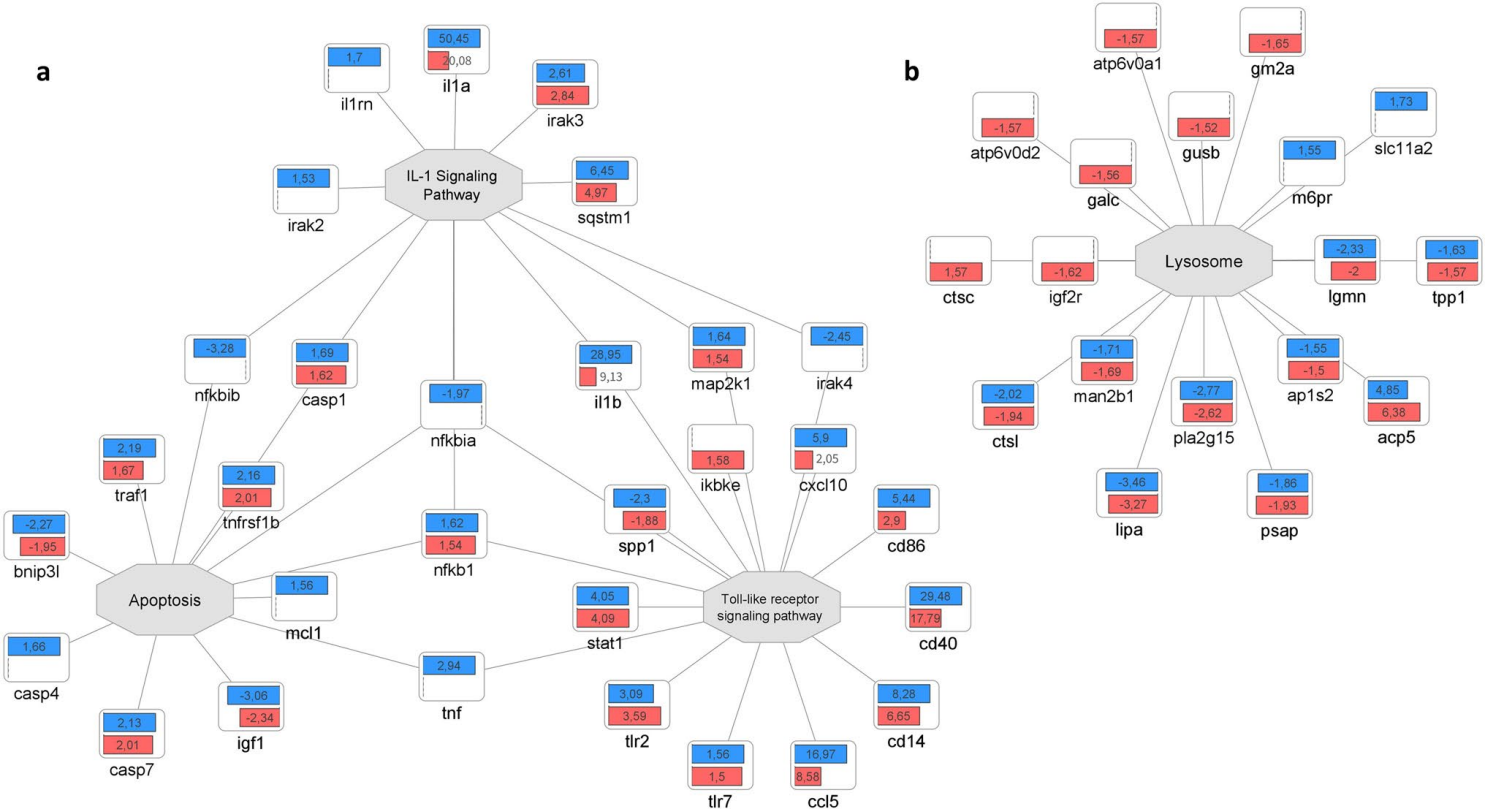
Quantitative comparison of the pro-inflammatory response elicited by PA57 and MSSA60-extracted exopolysaccharide in macrophages. Fold change quantitative data indicate that EPS extracted from PA57 (blue) and EPS extracted from MSSA60 (red) differ in terms of pro-inflammatory (**a**) and lysosomal (**b**) responses (color figure online)

**Fig. 11:**
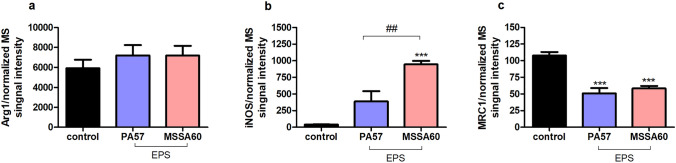
Quantitative comparison of the selected pro-inflammatory protein markers elicited by PA57 and MSSA60-extracted exopolysaccharide in macrophages. Normalized intensities of selected proteins: arginase-1 (**a**), iNOS (**b**), and MRC1 (**c**) measured by mass spectrometry evidence the M1-like response characteristics in macrophages exposed to bacterial EPS and indicate the difference between strains in the magnitude of pro-inflammatory activation. ***p < 0.001 vs. non-stimulated cells as negative control, ^##^*p* < 0.005

The examined bacteria strains exhibited qualitatively similar, but quantitatively different changes in the proteome of peritoneal macrophages (Fig. 6, suppl. Fig. S2). The majority of the identified regulated proteins were common between MSSA60 and PA57 (Fig. 6a); however, most of the PA57-specific proteins were functionally related to inflammatory response processes and cytokine signaling (Fig. 6b), indicating that macrophage activation was more pronounced in the PA57 group as compared to MSSA60 (Fig. 10). The latter notion is in line with the data regarding the classical macrophage phenotype markers assayed by mass spectrometry and immunoblotting, showing a more pronounced increase in iNOS concentration in the PA57 group as compared to MSSA60 (Fig. 11). Interestingly, our in-depth proteome analysis allowed for the identification of several differences between two bacterial strains. PA57-specific changes were related to mitochondrial oxidative phosphorylation, while the regulated proteins were collectively downregulated as compared to the control and MSSA60 groups (Figs. 6b, 8). On the contrary, MSSA60-specific protein changes were related to lysosomal proteins involved in the metabolism of sphingolipids and glycans and were repressed in activated cells (Fig. 6b).

Similarly to the whole bacteria, the strain-specific exopolysaccharide evoked a substantial macrophage response, as evidenced by its proteome changes. The differences between the origins of the EPS were more homogeneous than those observed for the whole bacteria (Fig. 9a) and included mainly the inflammatory and metabolic pathways (Fig. 9b). Intriguingly, in line with the data from the whole bacteria, PA57-derived EPS was more effective in inducing classical M1-like molecular features (i.e., TLRs signaling, IL-1α and IL-1β induction, aggravated apoptosis; Fig. 10a), while the downregulation of lysosomal proteins was biased toward the MSSA60-extracted EPS group (Fig. 10b).

## Discussion

Advanced severe lung CF is characterized by chronic inflammation and decline in lung function [26]. The major players of CF pathogenesis are biofilm-forming bacteria strains (*P. aeruginosa* and *S. aureus—*MSSA/MRSA) and defective cells of innate immunity (neutrophils and macrophages). It has been well documented that both these cells isolated from the CF environment are hyperinflammatory and compromised phagocytes [27]. Two causative factors are responsible for these altered properties: an intrinsic factor, a mutant CFTR gene expression in phagocytic cells, and the unique biofilm-formed environment. The latter, an acquired factor, is primarily linked to *P. aeruginosa*, the predominant pathogen infecting advanced CF patients. Importantly, it has been shown that *P. aeruginosa*, due to its plasticity and strong adaptive capacity, is able to establish chronic hyperinflammatory infections in CF [28].

In this study, interactions between the CF-derived bacterial strains and murine C57BL/6 leukocytes were investigated. Going more into the details, murine neutrophils and macrophages were stimulated in vitro either with killed bacterial cells or with components of bacterial cells (LPS, PG) and biofilm components (EPS, DNA). A murine model was chosen to exclude any influence of the intrinsic factor (CFTR defect) on the cells’ functions. To compare the effect of various CF pathogens, we selected the following strains: PA57 and MSSA60—the strong biofilm-forming strains, isolated from the same patient with severe CF; PA43 and MRSA75—the weak biofilm-forming strains isolated from two patients with mild CF. Importantly, to compare the pro-inflammatory capacity of biofilm matrix components (EPS, DNA) derived from distinct bacterial strains, we used them at the same effective concentrations that were established in the preliminary experiments. Obviously, in vivo, the strong EPS-PA57 is able to achieve higher concentration than the weak EPS from the PA43 strain and will have greater impact on hyperinflammatory response. Positive correlation between concentration of *P. aeruginosa* EPS and its stimulatory effect on pro-inflammatory, M1-type cytokine generation have been previously described by us [8].

Essentially, the results from our experimental system allow to formulate the following thesis.

- Neutrophils stimulated with *P. aeruginosa* and *S. aureus* isolated from the sputum of CF patients exhibit hyperinflammatory response.

C57BL/6 murine neutrophils, exposed in vitro with bacterial cells of the selected CF pathogens produced massive amount of pro-inflammatory cytokines, such as TNF-α, IL-6 and IL-12p40 and moderate amount of anti-inflammatory IL-10 and ROS (suppl. Fig. S2). However, PA strains (PA57, PA43) were much stronger inducers of IL-6 and IL-12p40 generation than both the SA strains. Notably, the ratio of IL-6 to anti-inflammatory IL-10 was markedly higher in PA57 than that of MSSA60-stimulated neutrophils (IL-6/IL-10: PA57 = 9.3; MSSA60 = 1.7). Interestingly, macrophages stimulated in the same conditions produced significantly less cytokines, especially IL-12p40. It is in keeping with other studies demonstrating the primary role of neutrophils in CF tissue damage and their responsibility for the decline in respiratory function [29].

- Macrophages stimulated with CF pathogens exert M1-like phenotype. *P. aeruginosa* is a dominant extrinsic stimulator of hyperinflammatory response of macrophages.

C57BL/6 macrophages stimulated with distinct CF PA strains generated a similar pattern of cytokines regardless of their biofilm-forming properties and their origin (PA57-severe vs PA43-mild CF). However, both tested mucoid PA strains were markedly stronger stimulators of cytokine generation than SA strains (MSSA60, MRSA75). Moreover, these results suggest that *P. aeruginosa* is a major player in the hyperinflammatory response of macrophages and their polarization into M1-type cells. To verify this thesis in further studies, we focused on a comparison of the inflammatory properties of PA57 and MSSA60, the pathogens isolated from the same patient with severe advanced CF. The patient was 14 years old, with FEV1 < 20% and long history of chronic, recurrent lung infections. Thus, both PA57 and MSSA60 clinical isolates might have the same opportunities to undergo evolutionary diversification and phenotype adaptation to the CF environment [30]. As a consequence of this evolution, the adapted strains have particular phenotypes that promote persistent infection, as mentioned by others [31]. However, the results from our studies, the pattern of inflammatory cytokine, and the expression of iNOS marker indicate that PA57 was a prominently stronger inducer of M1 markers than MSSA60, despite the fact that they shared the same CF environment. Therefore, it was reasonable to confirm this observation using a quantitative proteomic approach for the characterization of proteome-wide changes in macrophages exposed to these bacteria strains in vitro.

To verify and expand the molecular observations regarding different secretory properties of macrophages exposed to different bacteria strains and their biofilm components, we performed an in-depth proteome-wide assessment of the intracellular protein abundance changes. The findings not only confirm the profound inflammatory response elicited by both bacteria strains and their biofilm, but also strongly point to the notion that this hyperinflammatory response is more pronounced, both qualitatively and quantitatively, in macrophages exposed to biofilm-forming mucoid PA57. Several key pro-inflammatory markers related to the nuclear factor kappa-B (NF-κB) signaling pathway, including its subunits and stimulators (i.e., NFKB1, NFKB2, RELB, BCL10) as well as several effector proteins (i.e., iNOS, IL-1α, IL-1β, CD14, CD40, DDX58, NOX2) were markedly induced to a higher extent by PA57 compared to MSSA60. Importantly, these observations were consistent regardless of the type of stimuli (whole bacteria or their biofilm components). The above-mentioned differences between the bacterial strains were additionally reinforced by the changes in the abundance of several components of the mitochondrial respiratory chain complexes, which were all downregulated in stimulated macrophages but to a greater extent in the PA57 group. The metabolic switch from oxidative phosphorylation to aerobic glycolysis is a well-recognized hallmark of pro-inflammatory M1-like macrophage activation [32]. Taken together, both functional and metabolic responses provide evidence of differences in the magnitude of hyperinflammatory macrophage activation in response to PA57 and MSSA60 bacterial strains.

Macrophages can respond to microbial infections with programmed cell death (PCD), with apoptosis, pyroptosis, and necroptosis as the major pathways that are strictly regulated to ensure adequate immune response [33]. In contrast to apoptosis, other forms of controlled lytic death of macrophages, including pyroptosis and necroptosis, can induce the inflammatory reaction [34]. In our proteomic dataset, we identified the induction of several molecular features of PCD including key necroptosis/pyroptosis effectors: NLRP3, caspase-1, and caspase-8 as well as several members of signal transducer and activator of transcription proteins (STAT1, STAT2 and STAT3). Likewise, the induction of these molecules was more pronounced in the PA57 group compared to MSSA60. Furthermore, we identified the molecular fingerprint suggesting increased ferroptosis in stimulated cells with induction of heme oxygenase 1 (HO-1) as a key marker and regulator of the process [35]. Similarly to other PCD programs, ferroptosis seems to be more induced in PA57-stimulated macrophages. The latter notion is in line with clinical observations regarding the role of ferroptosis in cystic fibrosis induction and progression [36], where *P. aeruginosa* is recognized as an important opportunistic pathogen responsible for ferroptotic cell death in the airways [37]. Altogether, the proteomic data suggest that macrophages stimulated with PA57 rather than MSSA60 are more susceptible to initiate different programs of lytic form of death.

Intriguingly, our proteomic data point to lysosomes as the additional cellular compartment that is differentially regulated in response to PA57 and MSSA60 bacteria or their biofilm. Several lysosomal enzymes and proteases (i.e., CTSC, GALC, GUSB), lysosomal protein transporters (including IGF2R and GM2A), and vacuolar ATPase subunits (ATP6V0A1 and ATP6V0D2) are repressed to a greater extent in the MSSA60 group compared to the PA57 strain (Fig. 10). Interestingly, recent studies provide evidence that *Staphylococcus aureus* may escape autophagic degradation by inhibiting autophagy flux and compromising lysosomal function [38, 39]. The proposed mechanism was based on the activation of transcription factor EB (TFEB) that was enhanced early under *S. aureus* infection, which was followed by its accelerated degradation to ameliorate lysosomal functions due to the delayed activation of ERK, mTOR, and STAT3 [39]. Importantly, two of the TFEB-related genes that were affected in the process were *Atp6v0a1* and *Atp6v0d2*; thus, the ATPase components identified in our proteomic dataset as repressed in the MSSA60 group (Fig. 10). All of the above imply that macrophages stimulated with *Staphylococcus aureus* MSSA60 strain may exhibit a compromised phagocytotic ability in comparison with PA57 that may influence the CF onset due to the reduced clearance of all accompanying pathogens.

## Conclusions

The present study explicitly demonstrated the prominent role of clinical CF isolates of mucoid *P. aeruginosa* strains in the M-1 polarization of macrophages. We have previously proposed a new term for M1-like macrophages polarized by mucoid *P. aeruginosa* strains as BAMs [8]. Even though our stimulated C57BL/6 murine macrophages differ from CFTR-defected cystic fibrosis macrophages, both types of cells exert similar regulatory properties of inflammation [27]: namely, they (i) increase the secretion of pro-inflammatory mediators, (ii) sustain hyperinflammation, and (iii) have increased responsiveness to external stimuli. However, further studies, using an experimental model of CF, are necessary to explain the joint effect of *P. aeruginosa* stimulation and intrinsic genetic factor (CFTR defect) on the hyperinflammatory and phagocytic impact of macrophages. In a case of reduced defense properties of BAMs (inefficient bacterial killing and/or dampened efferocytosis), we suggest that their repolarization might be a new therapeutic strategy in a lung CF.

Finally, in spite of a tremendous improvement of our knowledge concerning the pathogenesis of CF, we still do not know what is the early pathological event in CF airways: neutrophilic inflammation or bacterial infection?

## Supplementary Information

Below is the link to the electronic supplementary material.

Supplementary file1 (PDF 138 KB)

Supplementary file2 (PDF 170 KB)

Supplementary file3 (PDF 90 KB)

Supplementary file4 (XLSX 160 KB)

## Data Availability

All data generated or analyzed during this study are included in this article. Further inquiries can be directed to the corresponding author.
