# Supplementary material for: Biofilm-forming strains of P. aeruginosa and S. aureus isolated from cystic fibrosis patients differently affect inflammatory phenotype of macrophages
Source: Inflamm Res. 2023 May 31;72(6):1275–89. doi: 10.1007/s00011-023-01743-x (PMC10279583; doi:10.1007/s00011-023-01743-x)
Supplement: Supplementary file 1 — Supplementary file1 (PDF 138 KB) [file 11_2023_1743_MOESM1_ESM.pdf]

Inflammation Research

Biofilm forming strains of *P. aeruginosa* and *S. aureus* isolated from cystic fibrosis patients differently affect inflammatory phenotype of macrophages.

Marta Ciszek-Lenda, Grzegorz Majka, Maciej Suski, Maria Walczewska, Sabina Górska, Edyta Golińska, Angelika Fedor, Andrzej Gamian, Rafał Olszanecki, Magdalena Strus and Janusz Marcinkiewicz

Corresponding Author:

Grzegorz Majka

Jagiellonian University Medical College,

Faculty of Medicine, Department of Immunology,

Czysta 18, 31-121 Krakow, Poland

Tel: +48126325865

E-mail: grzegorz.majka@uj.edu.pl

Supplementary Figure S1

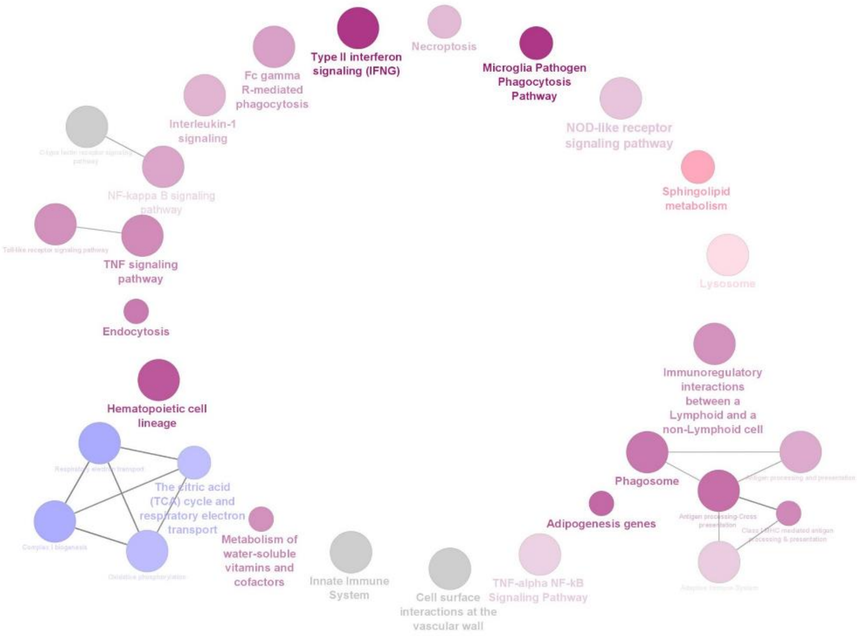

Supplementary Fig. S1 Functional grouping and pathway annotations of the differentially regulated proteins in PA57 and MSSA 60 activated macrophages. The functional grouping of regulated proteins in activated macrophages confirms similarities (violet) and highlights differences between the bacterial strains PA57 (blue) and MSSA60 (red)
