# Supplementary material for: Biofilm-forming strains of P. aeruginosa and S. aureus isolated from cystic fibrosis patients differently affect inflammatory phenotype of macrophages
Source: Inflamm Res. 2023 May 31;72(6):1275–89. doi: 10.1007/s00011-023-01743-x (PMC10279583; doi:10.1007/s00011-023-01743-x)
Supplement: Supplementary file 2 — Supplementary file2 (PDF 170 KB) [file 11_2023_1743_MOESM2_ESM.pdf]

Inflammation Research

Biofilm forming strains of *P. aeruginosa* and *S. aureus* isolated from cystic fibrosis patients differently affect inflammatory phenotype of macrophages.

Marta Ciszek-Lenda, Grzegorz Majka, Maciej Suski, Maria Walczewska, Sabina Górska, Edyta Golińska, Angelika Fedor, Andrzej Gamian, Rafał Olszanecki, Magdalena Strus and Janusz Marcinkiewicz

Corresponding Author:

Grzegorz Majka

Jagiellonian University Medical College,

Faculty of Medicine, Department of Immunology,

Czysta 18, 31-121 Krakow, Poland

Tel: +48126325865

E-mail: grzegorz.majka@uj.edu.pl

Supplementary Figure S2

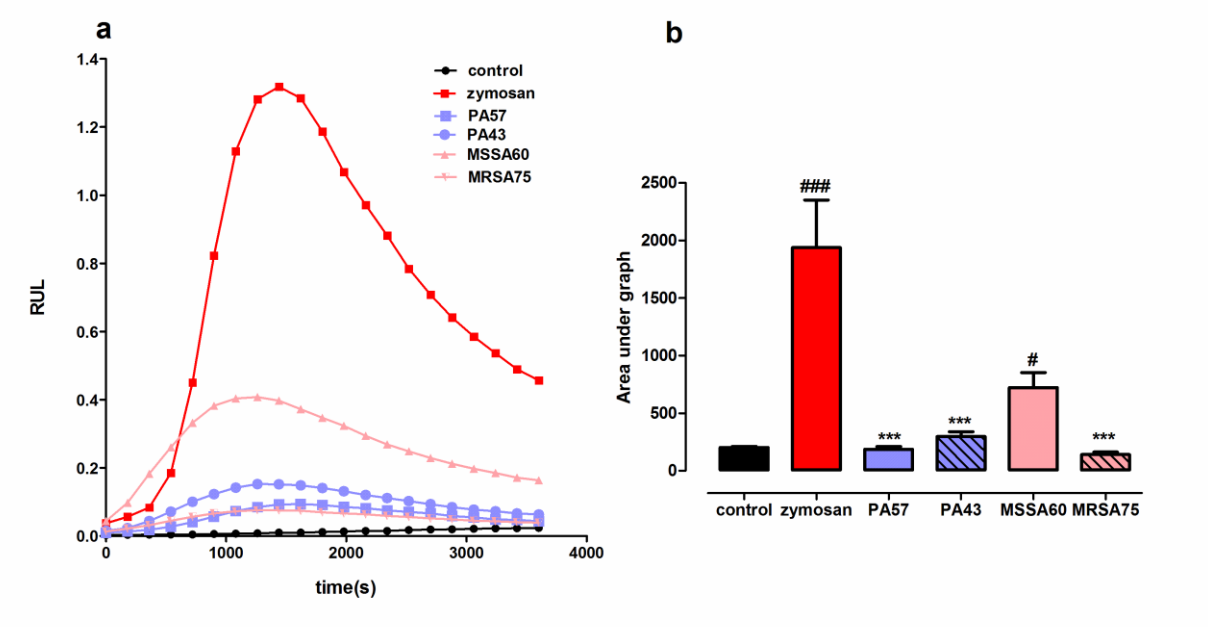

Supplementary Fig. S2 Effect of PA57, PA43, MSSA60, MRSA75 on ROS production by neutrophils. Cells (5×10^5/cells/well) were stimulated with tested bacterial strains or zymosan, as a positive control (0.2 µg/ml). Then, LCL was performed and measured as described in methods. Panel **a** shows one representative experiment, panel **b** shows mean ± SEM values of three independent experiments. *** p<0.001, vs. zymosan; #p < 0.05, ##p < 0.005, vs. non-stimulated cells as negative control, One-way ANOVA and Dunnett's as post hoc comparison test
