# Supplementary material for: Biofilm-forming strains of P. aeruginosa and S. aureus isolated from cystic fibrosis patients differently affect inflammatory phenotype of macrophages
Source: Inflamm Res. 2023 May 31;72(6):1275–89. doi: 10.1007/s00011-023-01743-x (PMC10279583; doi:10.1007/s00011-023-01743-x)
Supplement: Supplementary file 3 — Supplementary file3 (PDF 90 KB) [file 11_2023_1743_MOESM3_ESM.pdf]

Inflammation Research

Biofilm forming strains of *P. aeruginosa* and *S. aureus* isolated from cystic fibrosis patients differently affect inflammatory phenotype of macrophages.

Marta Ciszek-Lenda, Grzegorz Majka, Maciej Suski, Maria Walczewska, Sabina Górska, Edyta Golińska, Angelika Fedor, Andrzej Gamian, Rafał Olszanecki, Magdalena Strus and Janusz Marcinkiewicz

Corresponding Author:

Grzegorz Majka

Jagiellonian University Medical College,

Faculty of Medicine, Department of Immunology,

Czysta 18, 31-121 Krakow, Poland

Tel: +48126325865

E-mail: grzegorz.majka@uj.edu.pl

Supplementary Table S1. IL-6/IL-10 ratio* in supernatants from neutrophils and macrophages induced by tested bacteria

Neutrophils	**IL-6 : IL-10**	Macrophages	**IL-6 : IL-10**
control	2,8	control	0,51
PA57	9,3	PA57	7,59
PA43	11,07	PA43	9,87
MRSA75	0,58	MRSA75	18,41
MSSA60	1,72	MSSA60	6,62

*IL-6/IL-10 ratio was calculated from data shown in Figure 1 and 2, b and d.
